# Secure and Privacy-Preserving Body Sensor Data Collection and Query Scheme

**DOI:** 10.3390/s16020179

**Published:** 2016-02-01

**Authors:** Hui Zhu, Lijuan Gao, Hui Li

**Affiliations:** Key Laboratory of Integrated Services Networks, Xidian University, Xi’an 710071, China; gaolijuanxd@163.com (L.G.); lihui@mail.xidian.edu.cn (H.L.)

**Keywords:** body sensor network, privacy-preserving, data query, outsourced computing

## Abstract

With the development of body sensor networks and the pervasiveness of smart phones, different types of personal data can be collected in real time by body sensors, and the potential value of massive personal data has attracted considerable interest recently. However, the privacy issues of sensitive personal data are still challenging today. Aiming at these challenges, in this paper, we focus on the threats from telemetry interface and present a secure and privacy-preserving body sensor data collection and query scheme, named SPCQ, for outsourced computing. In the proposed SPCQ scheme, users’ personal information is collected by body sensors in different types and converted into multi-dimension data, and each dimension is converted into the form of a number and uploaded to the cloud server, which provides a secure, efficient and accurate data query service, while the privacy of sensitive personal information and users’ query data is guaranteed. Specifically, based on an improved homomorphic encryption technology over composite order group, we propose a special weighted Euclidean distance contrast algorithm (WEDC) for multi-dimension vectors over encrypted data. With the SPCQ scheme, the confidentiality of sensitive personal data, the privacy of data users’ queries and accurate query service can be achieved in the cloud server. Detailed analysis shows that SPCQ can resist various security threats from telemetry interface. In addition, we also implement SPCQ on an embedded device, smart phone and laptop with a real medical database, and extensive simulation results demonstrate that our proposed SPCQ scheme is highly efficient in terms of computation and communication costs.

## 1. Introduction

**Figure 1:**
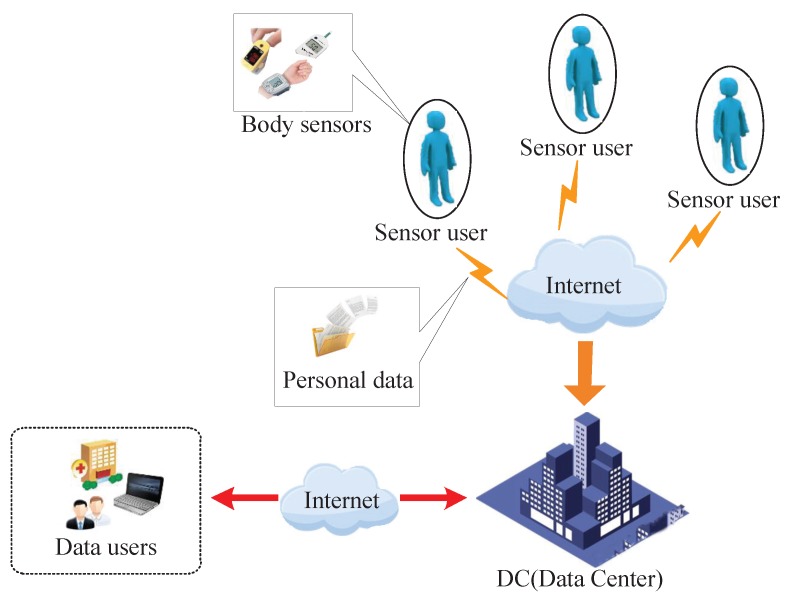
Body sensor data collection and query service scenario.

In recent years, with the popularization of wearable sensors and telemedicine, body sensor networks (BSN), which comprise multiple sensor nodes and a coordinator worn on a human body, can collect the personal information of the human body (such as heart rate, blood glucose and electrocardiogram) by sensor nodes [1,2,3,4,5,6]. The collected information first is delivered to the coordinator, then is forwarded to a remote server through a network interface for further processing [7,8]. As shown in Figure 1, vast quantities of the sensor users’ personal data are collected by body sensors and recorded by a data center per second. Since large-scale aggregate analysis of personal data can yield valuable results and insights, which can address public health challenges and provide new avenues for scientific discovery [9], data center trends toward providing on-demand data query service for users. However, this requires huge storage space and enormous computing resources, which are tremendous burdens on data centers. As the survey [10] shows that roughly 55 percent of respondents plan to use cloud services for analysis queries, cloud computing is a promising way to integrate personal data resources and to provide a uniform query service to researchers [11,12,13,14,15]. Since personal data are regarded as sensitive and private assets of sensor users, how to provide accurate data query services without revealing confidential personal data has attracted considerable interest recently.

To address these security and privacy issues, differential privacy [16], homomorphic encryption [17] and searchable encryption [18] are widely used. However, differential privacy cannot provide accurate query results for users; traditional homomorphic encryption schemes are time consuming and resource consuming; searchable encryption cannot provide query services over encryption data. Therefore, the above methods are not suitable for multi-dimension personal data query services.

Different from the methods discussed above, in this paper, we focus on the threats from telemetry interface [19,20] and propose a new secure and privacy-preserving body sensor data collection and query scheme, called SPCQ, for outsourced computing. In the proposed scheme, users’ personal information is collected by body sensors of different types and converted into multi-dimension data, and each dimension is converted into the form of a number and uploaded to the cloud server in ciphertext. After that, the cloud server provides query services to data users by the privacy-preserving weighted Euclidean distance contrast (WEDC) algorithm for multi-dimension vectors. Meanwhile, SPCQ can protect the confidentiality of sensor users’ personal data and the privacy of data users’ query, with low overheads in computation and communication.

The remainder of this paper is organized as follows. In Section 2, we review the related work. In Section 3, we define the system model and security model and identify our design goal. Additionally, in Section 4, we recall the bilinear pairing of the composite order, Euclidean distance and the 2DNF cryptosystem as the preliminaries. Then, we present our SPCQ scheme in Section 5, followed by the security analysis and performance evaluation in Section 6 and Section 7, respectively. Finally, we draw our conclusions in Section 8.

## 2. Related Work

In recent years, how to achieve operations over encrypted data has attracted considerable interest, and most of the proposed schemes are based on differential privacy, homomorphic encryption and searchable encryption.

The differential privacy notion was first formulated by Dwork [16], which can provide information about the database while simultaneously ensuring very high levels of privacy. Barthe *et al.* [21] presented CertiPriv, a machine-checked framework for reasoning about differential privacy built on top of the Coq proof assistant. The scheme provided a framework for fine-grained reasoning about an expressive class of confidentiality policies. Additionally, Tschantz *et al*. [22] presented the first results towards automated verification of source code for differentially-private interactive systems, which developed a formal probabilistic automaton model of differential privacy for systems by adapting prior work on differential privacy for functions. To achieve automated verification of distributed differential privacy, Eigner *et al*. [23] presented the framework by comprising a symbolic definition of differential privacy for distributed databases that takes into account Dolev–Yao intruders, and the scheme can overhear, intercept and synthesize the cryptographic messages exchanged on the network. However, the above differential privacy scheme cannot provide accurate query services because of added randomized noise.

Homomorphic encryption is a usual method to achieve data operations over encrypted data without decrypting it. Rivest *et al*. [17] first introduced homomorphism and presented four solutions to achieve homomorphic encryption. Then, Goldwasser *et al*. [24] proposed the first semantically-secure homomorphic encryption scheme, and many other additively homomorphic encryption schemes with proofs of semantic security [25,26,27] were presented. To achieve both additive and multiplicative homomorphisms, Gentry [28] designed a full homomorphic encryption scheme based on the mathematical object ideal lattices and uses the bootstrapping technique. It is semantically secure, and the security of the scheme is based on the split-key distinguishing problem. Then, other different full homomorphic encryption schemes were based on the elementary theory of algebraic number fields [29] and non-circuit [30]. However, most of these existing homomorphic encryption schemes have high time complexities, which is not suitable for practical use.

Keyword searchable encryption schemes usually build an encrypted searchable index, such that its content is hidden from the server unless it is given appropriate trapdoors generated via secret keys [31]. Song *et al*. [18] firstly studied searchable encryption in the symmetric key setting, and Boneh *et al*. [32] presented the first searchable encryption construction, where anyone with a public key can write to the data stored on the server, but only authorized users with a private key can search. However, public key solutions are usually very computationally expensive, and the keyword privacy could not be protected in the public key setting. To solve the multi-keyword ranked search over encrypted data problem, Cao *et al*. [33] proposed a basic idea of MRSE using secure inner product computation, and two improved MRSE schemes were given to achieve various stringent privacy requirements in two different threat models. However, searchable encryption can only provide keyword query rather than accurate computation query, which is not suitable for multi-dimension personal data.

Different from the above works, our proposed SPCQ scheme aims at the efficiency, accurate and privacy issues, and based on an improved homomorphic encryption technology over composite order group, we develop an efficient and privacy-preserving body sensor data collection and query scheme for outsourced computing. In particular, the proposed SPCQ can be easily implemented on different terminals, and the processing of the query is just needed in the cloud server. The computational costs in both the terminal and cloud server are acceptable.

## 3. Models and Design Goals

In this section, we define the system model, security requirements and identify our design goal.

### 3.1. System Model

**Figure 2:**
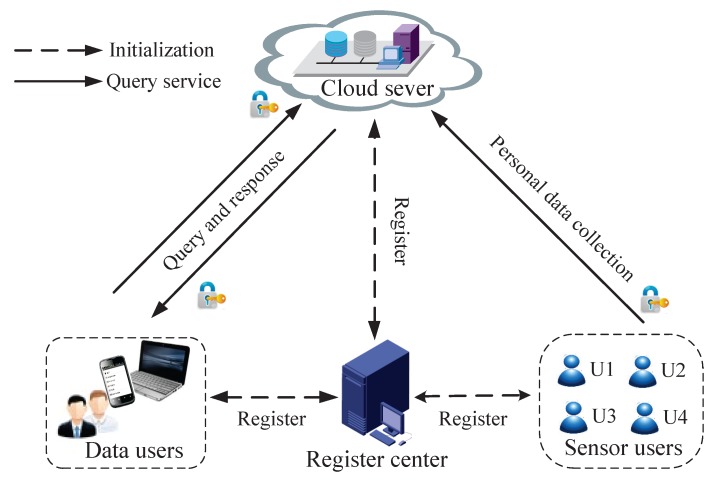
System model under consideration.

In our system model, we mainly focus on how to offer secure personal data collection and efficient query service over confidential personal data in the outsourced cloud server. Specifically, the system consists of four parts: *register center* (RC), *sensor user* (SU), *data user* (DU) and *cloud server*(CS), as shown in Figure 2.

- RC is a trusted third party, which bootstraps the system initialization by generating system parameters and providing a registration system for SU, DU and CS.
- SU collects real-time personal data by body sensors, and all SUs’ personal data will be uploaded to CS. To guarantee the data confidentiality, SU will perform some encryption operations before uploading data to CS.
- DU (e.g., the researcher), who is registered in RC, can send query request to CS for analysis of the accurate personal data items stored in it. To guarantee the privacy of DU’s query information, DU will perform some encryption operations during the process of the query. Meanwhile, SUs’ personal data should be kept secret from unauthorized users.
- CS is composed of many data storage nodes and computing nodes, stores more than a billion encrypted personal data items from SUs and provides accurate query services to DUs over encrypted personal data. CS mainly performs two functions: authentication and computing over encrypted data. The authentication component is used to check the identity of SUs and DUs, while the computing in encryption component is used to search and compute encrypted data items with DUs’ encrypted query request.

### 3.2. Security Requirements

The confidentiality of personal data from SUs and the privacy of DU’s query information are crucial for the success of a secure and privacy-preserving body sensor data collection and query scheme. In our security model, we consider CS is *honest-but-curious*. Specifically, CS faithfully executes the operations to search DUs’ demanded information over the encrypted personal data from SUs, but it also tries to analyze the query information and encrypted data to obtain users’ sensitive information. Therefore, in this paper, we focus on the threats from telemetry interface, and the following security requirements should be satisfied in a secure and privacy-preserving body sensor data collection and query scheme. Note that, in our current model, we do not consider that any two parties collude to disclose the third party’s privacy, *i.e.*, the collusion attack on privacy is beyond the scope of this work and will be discussed in future research.

- *Confidentiality*. SUs’ sensitive personal data should be kept secret from CS, *i.e.*, even if CS stores all personal data from SUs, it cannot identify any data item. In this circumstance, the confidentiality of the personal data can be guaranteed.
- *Privacy*. DU’s query information should be secrete from CS, *i.e.*, even if CS obtains all DUs’ queries and corresponding responses, it cannot identify DUs’ query information accurately. Additionally, other users (e.g., SUs and other DUs) cannot get any information of DU. In this circumstance, the privacy-preserving requirements of DU’s query information can be guaranteed. In addition, the privacy requirement also includes CS’s responses, *i.e.*, only legal DU can decrypt the corresponding response.
- *Authentication*. Authenticating an encrypted query that is really sent by a legal DU and has not been altered during the transmission, *i.e.*, if an illegal DU forges a query, this malicious operation should be detected, and only correct queries can be received by CS. The responses from CS should also be authenticated so that DUs can receive authentic and reliable query results. Moreover, the encrypted personal data from SUs can be authenticated by CS.

### 3.3. Design Goals

Under the aforementioned system model and security requirements, our design goal is to develop a secure and privacy-preserving body sensor data collection and query scheme for outsourced computing, which will provide secure personal data collection and storage for SUs and privacy-preserving accurate Euclidean distance query service for DUs. Specifically, the following three objects should be achieved.

- *The Security Requirements Should be Guaranteed.* If the personal data collection and query scheme does not consider the security, SUs’ data assets and DU’s actual query information could be disclosed. Then, the data collection and query service cannot jump in popularity. Therefore, the proposed scheme should achieve the confidentiality, privacy and authentication simultaneously.
- *A Personal Data query Service with High Accuracy Should be Guaranteed.* The user experience is one of the most critical aspects of data query service, and it is important that the precision of Euclidean distance query service cannot be lowered when protecting DU’s privacy. Therefore, the proposed scheme should also provide highly precise and reliable query service.
- *The Effectiveness in Computation and Communication Should be Achieved for Various Terminal Devices.* The personal data may be collected by different terminal devices, such as smart phone, embedded device, *etc*. Although the performance of terminal devices is continuously improved today, the battery is still limited. The proposed scheme should also consider the effectiveness in terms of computation and communication to reduce the power consumption of different terminals. Moreover, data users can access the data query service by mobile terminals, in order to lower the energy cost, the efficiency of the query service is very important. Furthermore, although CS is featured with high performance in storage and computation, since thousands of DUs will query the data at the same time, the efficiencies of computation and communication are still challenging.

## 4. Preliminaries

In this section, we recall the bilinear pairing technique, Euclidean distance and 2DNF cryptosystem, which serve as the basis of our proposed SPCQ scheme.

### 4.1. Bilinear Pairing of Composite Order

Let G and $G_{t}$ be two multiplicative cyclic groups of the same composite order $N=p_{1}\cdot p_{2}$ (where $p_{1}\;\;\text{and}\;\;\;p_{2}$ are big primes), and *g* is a generator of G. We suppose $e:G\times G\to G_{t}$ denotes the bilinear map (also referred to as a paring), which has the following properties.

(1) Bilinearity. $e\left(u^{a},v^{b}\right)=e{\left(u,v\right)}^{ab}$ holds for all u,v∈G and $a,b\in Z_{N}$;
(2) Non-degeneracy. $e\left(g,g\right)\neq 1_{G_{t}}$;
(3) Computability. For all u,v∈G,e(u,v) can be computed efficiently.

### 4.2. Euclidean Distance

Euclidean distance is a common definition of distance, it corresponds to the true distance of two points in *n*-dimensional space. The Euclidean distance between two points P and Q is the length of the line segment connecting them in Euclidean space. In Cartesian coordinates, if $P=(p_{1},p_{2},\ldots ,p_{n})$ and $Q=(q_{1},q_{2},\ldots ,q_{n})$ are two different *n*-dimensional vectors, then the Euclidean distance from P to Q or from Q to P is given by the Pythagorean formula:

$$d\left(P,Q\right)=\sqrt{{\left(q_{1}-p_{1}\right)}^{2}+{\left(q_{2}-p_{2}\right)}^{2}+\cdots +{\left(q_{n}-p_{n}\right)}^{2}}$$

To satisfy the practical circumstance and provide DUs with an accurate query service, we set different weight numbers $\left(w_{1},w_{2},\ldots ,w_{n}\right)$ for each dimension to form a weighted Euclidean distance:

$$d\left(P,Q\right)=\sqrt{w_{1}{\left(q_{1}-p_{1}\right)}^{2}+w_{2}{\left(q_{2}-p_{2}\right)}^{2}+\cdots +w_{n}{\left(q_{n}-p_{n}\right)}^{2}}$$

### 4.3. 2DNF Cryptosystem

The 2DNF [34] cryptosystem is a public-key system that can achieve the homomorphic properties, which resembles the Paillier [27] and Okamoto-Uchiyama [26] encryption schemes. Specially, the 2DNF cryptosystem consists of three sections: key generation, encryption and decryption.

- Key generation Gen(μ). Given a security parameter $\mu \in Z^{+}$, two μ−bit prime numbers $p_{1}$ and $p_{2}$ are first chosen, and $N=p_{1}\cdot p_{2}$ is computed. Two groups of the same order *N* are generated, *g* and *u* are two generators of G. Then, $h=u^{p_{2}}$ is computed as a random generator of G’s subgroup with order $p_{1}$. Finally, the public key $PK=(n,G,G_{t},e,g,h)$ and private key $SK=p_{1}$ are generated.
- Encryption. We assume the message space consists of integers in the set $\left\{0,1,\cdots ,T\right\}$ with $T<p_{2}$. Then, to encrypt a message *m* with public key *PK*, a random number *r* is selected from $\left\{0,1,\cdots ,N-1\right\}$, and the ciphertext $C=g^{m}h^{r}\in G$ is computed.
- Decryption. To decrypt ciphertext C with private key $SK=p_{1}$, notice that $C^{p_{1}}={\left(g^{m}h^{r}\right)}^{p_{1}}={\left(g^{p_{1}}\right)}^{m}$; let $\hat{g}=g^{p_{1}}$. To recover the corresponding message *m*, we need to compute the discrete log of $C^{p_{1}}={\hat{g}}^{m}$, where $\hat{g}=g^{p_{1}}$. Since 0≤m≤T, it only takes expected time $\hat{O}\left(\sqrt{T}\right)$ using Pollard’s lambda method [35] to get the message *m*.

Note that the decryption time in the system would be the polynomial time in the size of the message space $T_{s}$. Hence, it is obvious that the cryptosystem is efficiently suitable for short messages.

## 5. Proposed SPCQ

In this section, we present a secure and privacy-preserving body sensor data collection and query scheme for outsourced computing, which mainly consists of three phases: *system initialization*, *secure data collection* and *privacy-preserving query service*. For an easier expression, the definition of notations to be used in the proposed SPCQ scheme are shown in Table 1.

**Table 1:** Definition of notations in the proposed secure and privacy-preserving body sensor data collection and query (SPCQ) scheme.

Notation	Definition
*μ*	the system security parameter
$p_{1},p_{2}$	two big prime numbers
$N=p_{1}\cdot p_{2}$	the product of $p_{1}$ and $p_{2}$
$G,G_{t}$	the bilinear groups with order *N*
*e, g ,h*	the parameters of bilinear groups
$B_{1}$	$B_{1}=g^{p_{1}}$
$B_{2}$	$B_{2}=e{\left(g,g\right)}^{p_{1}}$
E()	the asymmetric encryption algorithm, *i.e.*, ECC
*H*()	the secure cryptographic hash function
HPS	the evaluation dataset
$\left(x_{i1},x_{i2},\ldots ,x_{in}\right)$	the feature parameters of a data item
$W=(w_{1},w_{2},\ldots ,w_{n})$	the weighted number of different dimensions
*d*	the weighted Euclidean search range of DU’s query
$F_{i}=\left(f_{i1},f_{i1}^{\prime },f_{i2},f_{i2}^{\prime },\ldots ,f_{in},f_{in}^{\prime }\right)$	the encrypted search index of a data item
$\langle q_{1},q_{1}^{\prime },\ldots ,q_{n},q_{n}^{\prime }\rangle$	DU’s encrypted query parameters

### 5.1. System Initialization

We consider RC as the trusted third party, which bootstraps the system. In the system initialization phase, RC first selects a security parameter *μ*, generates system parameters $\left(G,G_{t},p_{1},p_{2},e,g,h,N=p_{1}\cdot p_{2}\right)$ by executing Gen(μ) and calculates two secret bases $B_{1}=g^{p_{1}}$ and $B_{2}=e{\left(g,g\right)}^{p_{1}}$. Next, RC decides a multi-dimension weight vector $W=(w_{1},w_{2},\ldots ,w_{n})$ that each number denotes the weight value of the corresponding dimension. Then, RC picks a random number $r_{RC}\in Z_{N}^{*}$ as its private key $SK_{RC}$ and computes the corresponding public key $PK_{RC}=g^{SK_{RC}}$. In addition, RC determines an asymmetric cryptographic algorithm E(), *i.e.*, ECC, and a secure cryptographic hash function *H*(), where $H:{\left\{0,1\right\}}^{*}\to Z_{N}^{*}$ and $Z_{N}^{*}$ is a nonzero group of integer modulo *N*. Finally, RC publishes the system parameters as $\langle N,G,G_{T},e,g,h,PK_{RC},E\left(\right),H\left(\right)\rangle$ and keeps $\langle p_{1},SK_{RC}\rangle$ secretly.

When an SU or DU registers itself to RC, it picks a random number $r\in Z_{N}^{*}$ as the private key SK and computes and submits the corresponding public key $PK=g^{SK}$ to RC for the signature. Then, RC sends $\langle B_{1},B_{2},W\rangle$ to the registered SU and DU through a secure channel. Similarly, when CS registers itself, it generates the private and public key pair as $SK_{\mathrm{CS}}\in Z_{N}^{*}$, $PK_{CS}=g^{SK_{CS}}$, and submits $PK_{CS}$ to RC for the signature. After that, RC calculates $HP_{j}=H\left({B_{2}}^{j^{2}}\right)$, where 0≤j≤η and *η* is a big integer whose length is much less than 256 bits, and structures the set of data values $HPS=\left\{HP_{0},HP_{1},\ldots ,HP_{\eta }\right\}$. Then, RC ranks the dataset from the smallest to the largest and sends the ordered dataset HPS to CS. It is noteworthy that $\langle B_{1},B_{2}\rangle$ is not given to CS. After providing the registration function for SU, DU and CS, RC goes offline or suffers slowdowns against the single point of attack, since it has many secret parameters.

### 5.2. Secure Data Collection

SUs collect their real-time personal data through body sensors, and the data can be described by *n*-dimensional vectors $\left(x_{i1},x_{i2},\ldots ,x_{in}\right)$. Before uploading to CS, each data item in SU should be processed as follows.

- SU computes $x_{i1}^{\prime }=x_{i1}+H\left(B_{1}\right)$, $x_{i2}^{\prime }=x_{i2}+H\left(B_{1}\right),\ldots ,x_{in}^{\prime }=x_{in}+H\left(B_{1}\right)$, where $B_{1}$ is only known by registered SUs and DUs; this operation can resist the exhaustive attack.
- SU chooses *n* random numbers $r_{1},r_{2},\ldots ,r_{n}\in Z_{N}^{*}$ and computes the encrypted search index $F_{i}=\left(f_{i1},f_{i1}^{\prime },f_{i2},f_{i2}^{\prime },\ldots ,f_{in},f_{in}^{\prime }\right)$, which can be implicitly formed as follows.
  $$\begin{array}{l} \left\{\begin{array}{ccc} f_{i1}=B_{2}^{w_{1}\cdot {x_{i1}^{\prime }}^{2}} & & f_{i1}^{\prime }=g^{x_{i1}^{\prime }}\cdot h^{r_{1}}\\ f_{i2}=B_{2}^{w_{2}\cdot {x_{i2}^{\prime }}^{2}} & & f_{i2}^{\prime }=g^{x_{i2}^{\prime }}\cdot h^{r_{2}}\\ \vdots & & \vdots \\ f_{in}=B_{2}^{w_{n}\cdot {x_{in}^{\prime }}^{2}} & & f_{in}^{\prime }=g^{x_{in}^{\prime }}\cdot h^{r_{n}} \end{array}\right. \end{array}$$
- SU makes a signature $Sig=H{\left(F_{i}∥ID∥TS_{1}\right)}^{SK}$ using the private key SK, where $TS_{1}$ is the current timestamp to resist potential replay attack, and *ID* is the identify number of SU. Then, SU sends the signed data item $\langle F_{i}∥ID∥TS_{1}∥Sig\rangle$ to CS.
- After receiving the signed data item from SU, CS first checks the timestamp $TS_{1}$ and verifies the signature Sig by computing whether $e\left(g,Sig\right)=e(PK,H\left(F_{i}∥ID∥TS_{1}\right))$. If it does hold, the signature is accepted, since $e\left(g,Sig\right)=e{\left(g,H\left(F_{i}∥ID∥TS_{1}\right)\right)}^{SK}=e\left(PK,H\left(F_{i}∥ID∥TS_{1}\right)\right)$. Then, CS stores the data item $F_{i}$.

### 5.3. Privacy-Preserving Query Service

#### 5.3.1. User Query Generation

Registered DU $U_{j}$ is able to send a query request to CS without revealing his or her query information by the following steps.

- $U_{j}$ first decides a data item with *n* feature parameters $\left\{y_{1},y_{2},\ldots ,y_{n}\right\}$ that he or she is willing to query and computes $y_{1}^{\prime }=y_{1}+H\left(B_{1}\right),y_{2}^{\prime }=y_{2}+H\left(B_{1}\right),\ldots ,y_{n}^{\prime }=y_{n}+H\left(B_{1}\right)$ to increase the sample space.
- $U_{j}$ determines the weighted Euclidean distance search range *d* from the data item that he or she wants to query and computes encrypted query $\left(q_{1},q_{1}^{\prime },q_{2},q_{2}^{\prime },\ldots ,q_{n},q_{n}^{\prime }\right)$ as follows.
  $$\begin{array}{l} \left\{\begin{array}{lll} q_{1}=B_{2}^{w_{1}\cdot {y^{\prime }}_{1}^{2}-d^{2}} & & q_{1}^{\prime }=B_{1}^{2w_{1}\cdot {y^{\prime }}_{1}}\\ q_{2}=B_{2}^{w_{2}\cdot {y^{\prime }}_{2}^{2}} & & q_{2}^{\prime }=B_{1}^{2w_{2}\cdot {y^{\prime }}_{2}}\\ \vdots & & \vdots \\ q_{n}=B_{2}^{w_{n}\cdot {y^{\prime }}_{n}^{2}} & & q_{n}^{\prime }=B_{1}^{2w_{n}\cdot {y^{\prime }}_{n}} \end{array}\right. \end{array}$$
- $U_{j}$ uses the public key of CS $PK_{CS}$ to compute $Q=E_{PK_{CS}}(q_{1}\left|\right|q_{1}^{\prime }\left|\right|q_{2}\left|\right|q_{2}^{\prime }\left||\ldots |\right|q_{n}\left|\right|q_{n}^{\prime })$.
- $U_{j}$ makes a signature $Sig_{j}={\left(H\left(Q∥U_{j}∥TS_{2}\right)\right)}^{SK_{U_{j}}}$ using his or her private key $SK_{U_{j}}$, where $TS_{2}$ is the current timestamp to resist potential replay attack. Then, $U_{j}$ sends the encrypted data query request $\langle Q∥U_{j}∥TS_{2}∥Sig_{j}\rangle$ to CS.

#### 5.3.2. Search and Response

After receiving encrypted data query request $\langle Q∥U_{j}∥TS_{2}∥Sig_{j}\rangle$ from $U_{j}$, CS executes the following procedures to provide personal data query service.

- CS first checks the timestamp $TS_{2}$ and verifies the signature $Sig_{j}$ by computing whether $e\left(g,Sig_{j}\right)=e\left(PK_{DU_{j}},H\left(Q∥U_{j}∥TS_{2}\right)\right)$. If it does hold, the signature is accepted, since $e\left(g,Sig_{j}\right)=e{\left(g,H\left(Q∥U_{j}∥TS_{2}\right)\right)}^{SK_{U_{j}}}=e\left(PK_{U_{j}},H\left(Q∥U_{j}∥TS_{2}\right)\right)$.
- CS uses its secret key $SK_{CS}$ to decrypt *Q* and obtain $\langle q_{1},q_{1}^{\prime },q_{2},q_{2}^{\prime },\ldots ,q_{n},q_{n}^{\prime }\rangle$. Then, CS executes the proposed WEDC algorithm as follows.
  - For each data item $F_{i}$ stored in it, CS computes the search criteria $D_{i}$ as follows.
    $$\begin{array}{ll} D_{i} & =\frac{e\left(f_{i1}^{\prime },q_{1}^{\prime }\right)\cdot e\left(f_{i2}^{\prime },q_{2}^{\prime }\right)\cdot \;\ldots \;\cdot e\left(f_{in}^{\prime },q_{n}^{\prime }\right)}{f_{i1}\cdot f_{i2}\cdot \ldots \cdot f_{in}\cdot q_{1}\cdot q_{2}\cdot \ldots \cdot q_{n}}\\ & =\frac{e\left(g^{{x^{\prime }}_{i1}}h^{r_{1}},B_{1}^{2w_{1}\cdot {y^{\prime }}_{1}}\right)\cdot \;\ldots \;\cdot e\left(g^{{x^{\prime }}_{in}}h^{r_{n}},B_{1}^{2w_{n}\cdot {y^{\prime }}_{n}}\right)}{B_{2}^{w_{1}{{x^{\prime }}_{i1}}^{2}}\cdot \;\ldots \;\cdot B_{2}^{w_{n}{{x^{\prime }}_{in}}^{2}}\cdot B_{2}^{w_{1}{y^{\prime }}_{1}^{2}-d^{2}}\cdot \;\ldots \;\cdot B_{2}^{w_{n}{y^{\prime }}_{n}^{2}}}\\ & =\frac{e\left(g^{{x^{\prime }}_{i1}}h^{r_{1}},g^{p_{1}\cdot 2w_{1}\cdot {y^{\prime }}_{1}}\right)\cdot \;\ldots \;\cdot e\left(g^{{x^{\prime }}_{in}}h^{r_{n}},g^{p_{1}\cdot 2w_{n}\cdot {y^{\prime }}_{n}}\right)}{B_{2}^{w_{1}{{x^{\prime }}_{i1}}^{2}}\cdot \ldots \cdot B_{2}^{w_{n}{{x^{\prime }}_{in}}^{2}}\cdot B_{2}^{w_{1}{y^{\prime }}_{1}^{2}-d^{2}}\cdot \ldots \cdot B_{2}^{w_{n}{y^{\prime }}_{n}^{2}}}\\ & =\frac{e{\left(g,g\right)}^{p_{1}2w_{1}\cdot {x^{\prime }}_{i1}{y^{\prime }}_{1}}\cdot \ldots \cdot e{\left(g,g\right)}^{p_{1}\cdot 2w_{n}\cdot {x^{\prime }}_{in}{y^{\prime }}_{n}}}{B_{2}^{w_{1}{{x^{\prime }}_{i1}}^{2}}\cdot \ldots \cdot B_{2}^{w_{n}{{x^{\prime }}_{in}}^{2}}\cdot B_{2}^{w_{1}{y^{\prime }}_{1}^{2}-d^{2}}\cdot \ldots \cdot B_{2}^{w_{n}{y^{\prime }}_{n}^{2}}}\\ & =B_{2}^{d^{2}-\left(w_{1}{\left({x^{\prime }}_{i1}-{y^{\prime }}_{1}\right)}^{2}+\ldots +w_{n}{\left({x^{\prime }}_{in}-{y^{\prime }}_{n}\right)}^{2}\right)}\\ & =B_{2}^{d^{2}-\left(w_{1}{\left(x_{i1}-y_{1}\right)}^{2}+\ldots +w_{n}{\left(x_{in}-y_{n}\right)}^{2}\right)} \end{array}$$
  - CS computes $HD_{i}=H\left(D_{i}\right)$ and searches $HD_{i}$ within the evaluation dataset *HPS* by binary search algorithm to confirm whether $HD_{i}$ belongs to it. If $HD_{i}$ belongs to *HPS*, it means that data item $F_{i}$ satisfies DU’s query condition; add one to $M_{num}$, where $M_{num}$ denotes the number of data items that meets the query condition; otherwise, data item $F_{i}$ does not meet DU’s query condition.
  - After traversing through all data items, CS gets the number of data items that satisfy DU’s query condition $M_{num}$ and the number of all data items $N_{num}$, which can help DU to achieve the statistical query of the personal data. Then, CS encrypts $N_{num}$ and $M_{num}$ with the asymmetric encryption algorithm *E*() and the public key of $U_{j}$$PK_{U_{j}}$ and uses its private key to make a signature $Sig_{CS}=H{\left(E_{PK_{U_{j}}}\left(N_{num}∥M_{num}\right)∥TS_{3}\right)}^{SK_{CS}}$.
  - Finally, CS sends $\langle E_{PK_{U_{j}}}\left(N_{num}∥M_{num}\right)∥TS_{3}∥Sig_{CS}\rangle$ to $U_{j}$.

*Correctness of WEDC Algorithm.* Here, we prove that CS can provide the correct statistical query service for DUs by executing the WEDC algorithm. Specifically, taking a look at the exponential of search criteria $D_{i}={B_{2}}^{d^{2}-\left(w_{1}{\left(x_{i1}-y_{1}\right)}^{2}+\cdots +w_{n}{\left(x_{in}-y_{n}\right)}^{2}\right)}$, we know $B_{2}$ is the generator of a cyclic group with order $p_{2}$, which is selected larger than 512 bits, and $w_{1}{\left(x_{i1}-y_{1}\right)}^{2}+\cdots +w_{n}{\left(x_{in}-y_{n}\right)}^{2}$ is the square of the weighted Euclidean distance between DU’s query data and data item $F_{i}$. In addition, since search range *d* is usually less than 10,000, we can define *η* = 10, 000 and *η*^2^ = 100, 000, 000. Therefore, if $F_{i}$ meets DU’s query condition, *i.e.*, $0\leq d^{2}-\left(w_{1}{\left(x_{i1}-y_{1}\right)}^{2}+\cdots +w_{n}{\left(x_{in}-y_{n}\right)}^{2}\right)\leq d^{2}\leq 100,000,000$, the corresponding $H(D_{i})$ must be in *HPS*, and $F_{i}$ will be counted as eligible data item; otherwise, $d^{2}-\left(w_{1}{\left(x_{i1}-y_{1}\right)}^{2}+\cdots +w_{n}{\left(x_{in}-y_{n}\right)}^{2}\right)\leq 0$, and $D_{i}={B_{2}}^{d^{2}-\left(w_{1}{\left(x_{i1}-y_{1}\right)}^{2}+\cdots +w_{n}{\left(x_{in}-y_{n}\right)}^{2}\right)}={B_{2}}^{p_{2}+d^{2}-\left(w_{1}{\left(x_{i1}-y_{1}\right)}^{2}+\cdots +w_{n}{\left(x_{in}-y_{n}\right)}^{2}\right)}$; then the corresponding $H(D_{i})$ will not be in *HPS* since $p_{2}+d^{2}-\left(w_{1}{\left(x_{i1}-y_{1}\right)}^{2}+\cdots +w_{n}{\left(x_{in}-y_{n}\right)}^{2}\right)\gg 100,000,000$; meanwhile, $F_{i}$ will not be counted as an eligible data item. Through the method we have stated, CS can correctly judge whether $F_{i}$ satisfies DUs’ query condition by utilizing WEDC algorithm.

#### 5.3.3. Query Result Reading

After receiving $\langle E_{PK_{U_{j}}}\left(N_{num}∥M_{num}\right)∥TS_{3}∥Sig_{CS}\rangle$ from CS, $U_{j}$ checks $TS_{3}$ and the signature $Sig_{CS}$ by verifying whether $e\left(g,Sig_{CS}\right)=e\left(PK_{CS},H{\left(E_{PK_{U_{j}}}\left(N_{num}∥M_{num}\right)∥TS_{3}\right)}^{SK_{CS}}\right)$ holds. Then, $U_{j}$ decrypts $E_{PK_{U_{j}}}\left(N_{num}∥M_{num}\right)$ with $SK_{U_{j}}$ to obtain the query result.

## 6. Security Analysis

In this section, we analyze the security properties of the proposed SPCQ scheme. Specifically, following the security requirements discussed earlier, our analysis will focus on how the proposed SPCQ scheme can achieve personal data confidentiality, DU’s query information privacy and source authentication of the personal data, query request and response.

- *The Proposed SPCQ Can Achieve Confidential Personal Data.* In our proposed SPCQ, personal data are secret from CS and DUs, although CS stores all encrypted data items and receives all query requests. First, since feature parameters $\left(x_{i1},x_{i2},\ldots ,x_{in}\right)$ collected by body sensors usually cover a smaller scope, to avoid the exhaustive attack against $\left(x_{i1},x_{i2},\ldots ,x_{in}\right)$ by Pollard’s lambda method, feature parameters are disturbed by calculating $x_{i1}^{\prime }=x_{i1}+H\left(B_{1}\right),x_{i2}^{\prime }=x_{i2}+H\left(B_{1}\right),\ldots ,x_{in}^{\prime }=x_{in}+H\left(B_{1}\right)$. In this way, the sample space is increased to more than 512 bits, which can prevent exhaustive attack efficiently. Before uploading to CS, feature parameters are encrypted to corresponding search index $\left(f_{i1},f_{i1}^{\prime },f_{i2},f_{i2}^{\prime },\ldots ,f_{in},f_{in}^{\prime }\right)$ by computing $f_{i1}=B_{2}^{w_{1}\cdot {x^{\prime }}_{i1}^{2}},f_{i1}^{\prime }=g^{{x^{\prime }}_{i1}}\cdot h^{r_{1}}$, *etc*., where $r_{1}$ is a random number to guarantee that for the same feature parameter, different SUs can obtain different search indexes. The above operations can achieve data perturbation and substitution and prevent CS from directly accessing SUs’ personal data. Moreover, to avoid the guessing attacks for $B_{2}$ in the evaluation dataset *HPS*, the relationship between $B_{2}$ and $HP_{j}$ is hidden by a secure hash function *H*(), where $HP_{j}=H\left(B_{2}^{j^{2}}\right)$. Therefore, from the above three aspects, CS cannot obtain the feature parameters of personal data according to uploaded data items. In addition, since DUs only can get the query statistic result from CS, SUs’ personal data are secret from DUs.
- *DU’s Query Information is Privacy-Preserving in the Proposed SPCQ.* In our proposed SPCQ, similarly, DU’s query condition is encrypted before being sent to CS. Specifically, DU’s query information $y_{1},y_{2},\ldots ,y_{n}$ is disturbed by calculating $y_{1}^{\prime }=y_{1}+H\left(B_{1}\right),y_{2}^{\prime }=y_{2}+H\left(B_{1}\right),\ldots ,y_{n}^{\prime }=y_{n}+H\left(B_{1}\right),$ which can resist the exhaustive attack by Pollard’s lambda method. Then, the query condition is encrypted by calculating $q_{1}=B_{2}^{w_{1}\cdot {y^{\prime }}_{1}^{2}-d^{2}},{q^{\prime }}_{1}={B_{1}}^{2w_{1}\cdot {y^{\prime }}_{1}},q_{2}=B_{2}^{w_{2}\cdot {y^{\prime }}_{2}^{2}},q_{2}^{\prime }={B_{1}}^{2w_{2}\cdot {y^{\prime }}_{2}},\ldots ,q_{n}=B_{2}^{w_{n}\cdot {y^{\prime }}_{n}^{2}},q_{n}^{\prime }={B_{1}}^{2w_{n}\cdot {y^{\prime }}_{n}}$, which can prevent CS from directly accessing the query data item and search range *d*. Since $B_{1}$ and $B_{2}$ are only known by SUs and DUs, and the collusion attack is not considered in the current security model, CS cannot obtain query information from the query request during the query process. Specifically, encrypted request and encrypted data are computed in CS to obtain the result, which will be sent back to DU, and CS also cannot obtain any useful information of DUs’ queries, even in the continuous search queries environment. Meanwhile, CS still can provide accurate query service to DUs by the proposed WEDC algorithm. Concretely, CS traverses all stored data items to compute the search criteria $D_{i}$ and find out all data items that satisfy the query condition, then it achieves the query statistic result and sends it to DU. It is notable that the result does not have particular meaning without any other useful information of DUs’ queries. Moreover, SUs are not involved in the query process, and DU’s query request is encrypted by CS’s public key $PK_{CS}$ before being sent to CS, so SUs cannot get DU’s query information even if they steal the request by eavesdropping. In addition, the response is encrypted by DU’s public key before being sent by CS, and thus, SUs and other registered DUs cannot decrypt the response. Therefore, from the above four aspects, DU’s query information is privacy-preserving in the proposed SPCQ.
- *The Authentication of the Personal Data, Query Request and Response are Achieved in the Proposed SPCQ.* In the proposed SPCQ, personal data from SUs, registered DU’s query request and the response of CS are signed by the BLS [36] short signature. Since the BLS short signature is provably secure under the CDH problem in the random oracle model, the source authentication can be guaranteed. Specifically, personal data from SU is signed by computing $Sig=H(F_{i}{∥ID∥TS_{1})}^{SK}$, where $TS_{1}$ is the current timestamp to resist potential replay attack and *SK* is SU’s private key to make sure only itself can make the signature. After receiving the signed data item, CS computes whether $e\left(g,Sig\right)=e(PK,H(F_{i}∥ID∥TS_{1}))$ to verify the source of the signature. Similarly, the registered DU’s query request and the response of CS are signed by the above operations. Moreover, since the unregistered user (such as SU and DU) does not have secret keys $B_{1}$ and $B_{2}$, he or she cannot upload personal data item or submit valid query request to CS. Therefore, personal data and the query request from the unregistered user and the response from the mendacious CS can be detected in the proposed SPCQ.

From the above analyses, we can conclude that SPCQ is secure and privacy-preserving and can achieve our security design goal.

## 7. Performance Evaluation

In this section, we evaluate the performance of our proposed SPCQ scheme in terms of the computation complexity of SU, DU and CS. In order to measure the integrated performance of SPCQ in a real environment, we also implement SPCQ on an embedded device, smart phone and laptop with a real medical database in a wireless network, by using a custom simulator built in JAVA. Specifically, an embedded device with a 650-MHz dual-core processor, a smart phone with a 1.4-GHz quad-core processor, 2 GB RAM, Android 4.0, and a laptop with a 2.0-GHz 4-core processor, 8 GB RAM, are chosen to simulate SU, DU and CS. Based on our proposed SPCQ scheme, a personal data gathering application is installed on the embedded device to simulate SU; a personal data query application built by JAVA, named SPCQ.apk, is installed on the smart phone to simulate DU; and simulators of CS are deployed in a laptop. In order to evaluate SPCQ in a real environment, a diabetes database [37], which has 100,000 items with 55 attributes, is selected as the data source, and the corresponding storage space is 692.37 MB. Meanwhile, an evaluation dataset *HPS* with 10,000 preprocessed SHA-256 values is constructed, which just needs a 312.5-KB storage space. In addition, we define $p_{1}$ and $p_{2}$ as 512-bit prime numbers, and $\eta^{2}$ as 100,000,000.

### 7.1. Computation and Communication Costs

The proposed SPCQ scheme can achieve effective personal data query service for CS and DUs. Specifically, we assume the dimension of each personal data item is *n*, and SU needs *n* multiplication operations and 3n exponentiation operations for each personal data item. When a DU $U_{j}$ generates an encrypted query $\left(q_{1},q_{1}^{\prime },q_{2},q_{2}^{\prime },\ldots ,q_{n},q_{n}^{\prime }\right)$, it requires *2n* exponentiation operations in $Z_{p_{2}}$. After receiving the query from $U_{j}$, CS firstly computes the search criteria $D_{i}$ for each data item $F_{i}$ stored in it, which takes n∗N pairing operations and 2n∗N multiplication operations for checking *N* resource items. After receiving the response from CS, $U_{j}$ decrypts the query statistic result with asymmetrical encryption algorithm, which is considered negligible compared to exponentiation and pairing operations. Denote the computational costs of an exponentiation operation in $Z_{p_{2}}$/$Z_{N^{2}}$, a multiplication operation in G/$G_{t}$/$Z_{N^{2}}$ and a pairing operation by $C_{e}$, $C_{m}$ and $C_{p}$, respectively. Then, for SU, DU and CS, the computational costs are $3n*C_{e}+n*C_{m}$, $2n*C_{e}$ and $nN*C_{p}+2nN*C_{m}$ in the proposed SPCQ.

Different from other time-consuming encryption techniques, the proposed SPCQ uses improved homomorphic encryption technology over a composite order group, which can provide accurate personal data query service and largely reduce the encryption time for the smart phone. In the following, for the comparison with SPCQ, we selected a privacy-preserving range query scheme (PPRQ) [38], which can only provide a one-dimensional range query service to DUs. Let *m* be the bit length of the attribute values, and the computational costs of SU, DU and CS are $2n*C_{e}+n*C_{m}$, $4m*C_{e}+2m*C_{m}$ and $23mnN*C_{e}+23mnN*C_{m}$, respectively.

Due to the factor of our proposed SPCQ, we take an *n*-dimension query into consideration. Then, we present the computation complexity comparison of the proposed SPCQ and PPRQ in Table 2, and it is obvious that our proposed SPCQ can achieve a privacy-preserving personal data query service with low computation complexity in DU and CS.

**Table 2:** Comparison of computation complexity.

Phase of Scheme	SPCQ	PPRQ
SU	$3n*C_{e}+n*C_{m}$	$2n*C_{e}+n*C_{m}$
DU	$2n*C_{e}$	$4m*C_{e}+2m*C_{m}$
CS	$nN*C_{p}+2nN*C_{m}$	$23mnN*C_{e}+23mnN*C_{m}$

**Figure 3:**
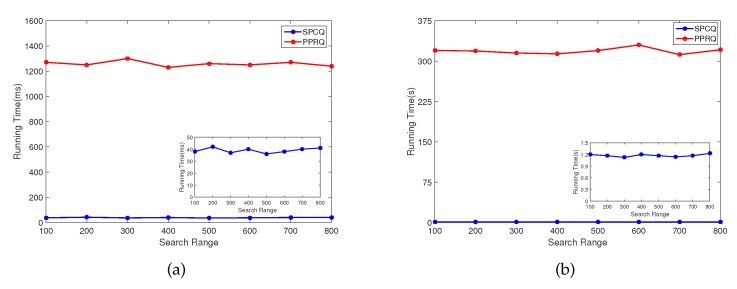
Computational overheads of SPCQ and PPRQ. (**a**) Average running time in DU with different search ranges; (**b**) average running time in CS with different search ranges.

For better comparison, we have implemented SPCQ and PPRQ in JAVA. In Figure 3a,b, we have plotted the computational overheads of SPCQ and PPRQ varying with different search ranges in DU and CS. From the two figures, we can see that in both DU and CS, the computational overheads of SPCQ and PPRQ vary slightly by increasing the search range, while the overheads of PPRQ are much higher than those of our proposed SPCQ scheme. It can be obviously shown that the SPCQ scheme largely reduces the computational complexity in DU and CS.

In addition, we have made the comparison of communication costs between SPCQ and PPRQ, as shown in Table 3. In SPCQ, the communication length in DU is 164∗n bytes, which is much less than that of PPRQ, whose communication length in DU is 512∗n bytes; the communication length in CS is 256 bytes, while that of PPRQ in CS is 1024∗N bytes; in addition, two times of communications between DU and CS are needed in both SPCQ and PPRQ. As we mentioned above, SPCQ is more efficient than PPRQ in terms of communication costs.

**Table 3:** Comparison of communication costs.

Phase of Scheme	SPCQ	PPRQ
Communication length in DU	164 ∗ *n* bytes	512 ∗ *n* bytes
Communication length in CS	256 bytes	1024 ∗ *n* bytes
Communication times	2	2

### 7.2. Simulation and Evaluation

To have a better evaluation of our proposed SPCQ, we analyze the factors that affect the computational costs of SU, DU and CS in detail. In addition, we evaluate the integrated performance of SPCQ.

#### 7.2.1. SU

**Figure 4:**
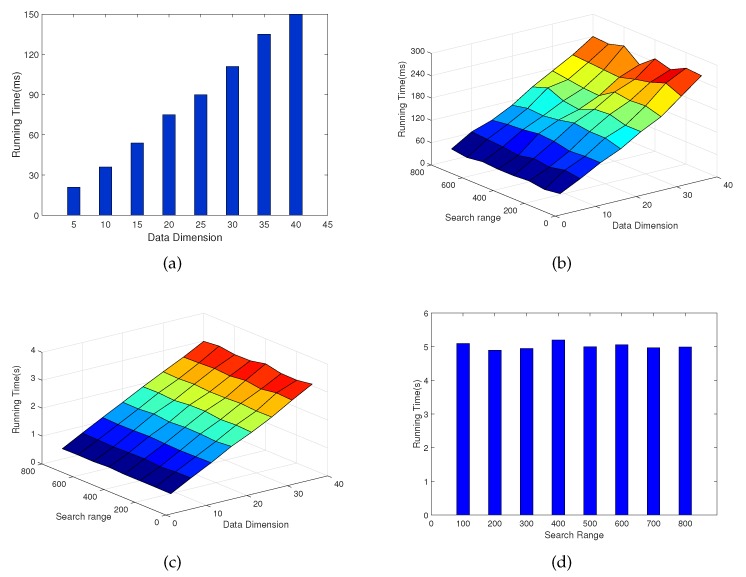
Computational cost of SPCQ. (**a**) Computational cost of SU in data collection; (**b**) computational cost of DU in query generation; (**c**) computational cost of CS with different search ranges and dimensions; (**d**) query response time in a real environment.

In our proposed SPCQ scheme, SUs collect their real-time personal data from body sensors and upload these data to CS per certain period. Before being sent to CS, the gathered personal data should be operated to obtain $\left(f_{i1},f_{i1}^{\prime },f_{i2},f_{i2}^{\prime },\ldots ,f_{in},f_{in}^{\prime }\right)$. Therefore, we have chosen different dimensions of personal data to illustrate the SU’s computational cost on the embedded device. As shown in Figure 4a, the dimensions of collected personal data are chosen from 5 to 40, and the average computational cost increases linearly with the increase of the dimension. Meanwhile, the computation on the embedded device is less than 150 milliseconds, which is acceptable for SU.

#### 7.2.2. DU

The query response time of DU (*i.e.*, smart phone) is an important aspect for our proposed SPCQ scheme, and the computational operations in the smart phone are query generation and result reading. Since the result reading only requires DU to decrypt the query result, which is negligible, therefore we have chosen different dimensions of the query request and different search ranges to illustrate DU’s computational cost. To observe the computational cost of the smart phone, the dimensions of each query are chosen from 5 to 40, and the search ranges are chosen from 100 to 800. Each condition is executed 100 times, and we have calculated the average time for different dimensions of the query request and search range. As shown in Figure 4b, the average computational cost increases linearly with the increase of the dimension, and it is nearly the same with different search ranges. The reason is that, when the smart phone generates a query request, it computes encrypted query parameters with the query condition. For the query condition with a high dimension, it takes more time to get the request, while different search ranges do not affect the computational cost.

#### 7.2.3. CS

In our proposed SPCQ scheme, after receiving a query request from DU, CS will compute the search criteria $D_{i}$ for each data item it stored, by using bilinear pairing over the composite order group, which is the main computation overhead of CS, *i.e.*, the efficiency of CS is impacted by the number of encrypted data resources, the dimension of each data item and the search range *d*. It is obvious that the computational cost in CS is increased with the number of encrypted data resources. Therefore, we have chosen different dimensions of data resources and different search ranges to illustrate the computational cost. As shown in Figure 4c, the dimensions of data resources are selected from 5 to 40, and eight search ranges of DU’s requests are selected from 100 to 800. We can learn from the figure that the computational cost of CS is nearly the same with different search ranges; meanwhile, the computational cost increases linearly with the increase of the data resource’s dimension.

#### 7.2.4. Integrated Performance in a Real Environment

In order to evaluate the integrated performance of our proposed scheme, SPCQ is deployed in a real environment with the real medical database mentioned above. Specifically, we have chosen 1000 items from the diabetes database, and the information of resources and corresponding encryption information is stored in CS, respectively. In addition, the smart phone and CS are connected through an 802.11g WLAN, and when DUs input the query data item and search range by SPCQ.apk, the smart phone will send a query request to CS and get the response through WLAN. We have run 100 times to evaluate the performance of SPCQ with eight search ranges (from 100 to 800), as shown in Figure 4d, the runtime is about five seconds, which is acceptable in a real environment.

## 8. Conclusions

In this paper, we have proposed a secure and privacy-preserving body sensor data collection and query scheme, called SPCQ, for outsourced computing. Based on an improved homomorphic encryption technology over composite order group, the proposed SPCQ scheme can achieve the confidentiality of SUs’ personal data and privacy-preserving of DU’s query information. Specifically, SUs’ personal data are collected in the form of multi-dimension vectors and uploaded to CS in ciphertext, and the data query request from the registered DU can be directly performed over ciphertext in CS, then the query result only can be decrypted by the registered DU. Therefore, DU can get an accurate query result without divulging his or her query information. Detailed security analysis shows its security strength and privacy-preserving ability, and extensive experiments are conducted to demonstrate its efficiency.
